# Mapping of the EQ-5D index from clinical outcome measures and demographic variables in patients with coronary heart disease

**DOI:** 10.1186/1477-7525-8-54

**Published:** 2010-06-04

**Authors:** Kimberley A Goldsmith, Matthew T Dyer, Martin J Buxton, Linda D Sharples

**Affiliations:** 1 Papworth Hospital NHS Trust, Cambridge, UK; 2 MRC Biostatistics Unit, Institute of Public Health, Cambridge, UK; 3 Institute of Psychiatry, King's College London, London, UK; 4 Health Economics Research Group, Brunel University, Uxbridge, UK; 5 National Collaborating Centre for Mental Health, The Royal College of Psychiatrists, London, UK

## Abstract

**Background:**

The EuroQoL 5D (EQ-5D) is a questionnaire that provides a measure of utility for cost-effectiveness analysis. The EQ-5D has been widely used in many patient groups, including those with coronary heart disease. Studies often require patients to complete many questionnaires and the EQ-5D may not be gathered. This study aimed to assess whether demographic and clinical outcome variables, including scores from a disease specific measure, the Seattle Angina Questionnaire (SAQ), could be used to predict, or map, the EQ-5D index value where it is not available.

**Methods:**

Patient-level data from 5 studies of cardiac interventions were used. The data were split into two groups - approximately 60% of the data were used as an estimation dataset for building models, and 40% were used as a validation dataset. Forward ordinary least squares linear regression methods and measures of prediction error were used to build a model to map to the EQ-5D index. Age, sex, a proxy measure of disease stage, Canadian Cardiovascular Society (CCS) angina severity class, treadmill exercise time (ETT) and scales of the SAQ were examined.

**Results:**

The exertional capacity (ECS), disease perception (DPS) and anginal frequency scales (AFS) of the SAQ were the strongest predictors of the EQ-5D index and gave the smallest root mean square errors. A final model was chosen with age, gender, disease stage and the ECS, DPS and AFS scales of the SAQ. ETT and CCS did not improve prediction in the presence of the SAQ scales. Bland-Altman agreement between predicted and observed EQ-5D index values was reasonable for values greater than 0.4, but below this level predicted values were higher than observed. The 95% limits of agreement were wide (-0.34, 0.33).

**Conclusions:**

Mapping of the EQ-5D index in cardiac patients from demographics and commonly measured cardiac outcome variables is possible; however, prediction for values of the EQ-5D index below 0.4 was not accurate. The newly designed 5-level version of the EQ-5D with its increased ability to discriminate health states may improve prediction of EQ-5D index values.

## Background

The EuroQoL 5D (EQ-5D) is a widely used generic measure of health related quality of life (HRQoL) and can be used to generate a single index value or utility [1-3]. This utility value is used for the calculation of quality-adjusted life years (QALYs) for cost-effectiveness analysis. The EQ-5D is currently recommended by the UK's National Institute for Health and Clinical Excellence (NICE) as a tool for quantifying utility in adults [3,4]. Quality of life and cost-effectiveness analyses are important for trials of interventions in cardiac patients and the EQ-5D has been used to calculate QALYs for cost-effectiveness analyses in several such trials [5-9].

Patients participating in clinical trials and other studies often have to complete many questionnaires, sometimes at multiple points in time. The EQ-5D is a short survey that has been shown to have good acceptability and feasibility in the general public and in cardiac patients [10-12]. However, in many studies it may not have been administered, for reasons of perceived patient burden from multiple questionnaires or because the study has not initially focused on economic questions. With the growing importance of cost-effectiveness estimation to inform Government and health insurers' policy decisions, it would be useful to be able to predict, or map, the EQ-5D index from other commonly collected demographics and clinical outcome variables.

Mapping of preference based measures using non-preference based tools is a growing area of study [13]. Such models could be used to predict the EQ-5D index in cases where it was not administered. Mapping of the EQ-5D index requires development of multiple variable regression models that predict the EQ-5D index with the minimum amount of error possible, so that predicted values give a reasonable estimate of the unobserved EQ-5D index. Mapping models may need to be derived separately for different disease groups, since the most effective predictors may vary between diseases. Also, mapping models need to incorporate variables that are commonly measured when studying the disease in question. For example, in studies of cardiac interventions, demographics and one or more common cardiac outcome measures, such as treadmill exercise time (ETT), Canadian Cardiovascular Society Angina Classification (CCS) and the Seattle Angina Questionnaire (SAQ), are generally gathered. Such variables would be obvious candidates for inclusion in models for mapping the EQ-5D index in cardiac patients.

Consistency in relationships between the EQ-5D index, patient characteristics and cardiac outcome measures across different studies/disease severity groups have recently been assessed using both aggregate and patient level data by our group [7,14]. The study using patient level data looked at the individual relationship between each of the cardiac measures described above and the EQ-5D index using data from several studies. Type of treatment and study variables were included to adjust for disease severity and type of population (ie. those selected for a clinical trial versus those entered into a cohort study) in order to get more accurate estimates of the magnitude of the relationship between the measures and the EQ-5D index. In the current study, the aim was to take these clinical measures in combination in a single model to predict the EQ-5D index. In this case, disease severity was taken into account using a single variable, and more implicitly from the point of view of stage of disease, as we felt this would be an important contributor to accurately predicting the EQ-5D index. The previous study found the relationship between the cardiac measures and the EQ-5D index were of different magnitudes and differed across patients having different treatments [14]. The treatments patients have roughly correspond to their disease severity, so it was important to take the disease stage into account when trying to map from disease specific variables to the EQ-5D index.

Several studies have looked at mapping using other generic or disease-specific HRQoL measures, with one other using clinical measures to map to the EQ-5D index [13,15]. This study aimed to use individual patient data to derive mapping models for the EQ-5D index in cardiac patients with different levels of disease severity by incorporating into these models multiple demographic factors and clinical cardiac measures commonly used when treating and studying these patients.

## Methods

### Data

The authors had access to individual patient data from 5 major studies in patients with cardiovascular disease in which both the EQ-5D and one or more commonly-used cardiac measurements were available, which were a subset of the studies used in our previous study [14]. A main dataset was created using data measured at multiple time points on patients participating in 4 randomised clinical trials [5,6,8,16], and 1 cohort study [17]. The studies covered diagnosis of cardiac disease and interventions in patients ranging from early disease managed medically to end-stage heart failure and are described briefly in Table 1. Measurements in the different studies were divided into baseline and post-treatment measurements and these were used as separate records to provide information about patient variables at different stages of disease. Further details of the studies used, the clinical measures, the use of measurements from different time points, and the individual relationship between each of these clinical measures and the EQ-5D index can be found in our earlier paper [14]. The dataset was then divided in two by taking a random sample of 60% of the data and separating that data from the remaining 40% to provide an estimation dataset and a validation dataset, respectively. There were similar proportions of records from each study in each of the two datasets (Table 1).

**Table 1:** Distribution of records selected for estimation and validation of models by study

Study	n (%) in 60% estimation dataset	n (%) in 40% validation dataset
CeCAT - Cost-effectiveness of functional cardiac testing in the diagnosis and management of CHD [8]	1061 (37.2)	664 (35.2)

ACRE - Appropriateness for coronary revascularization [17]	1449 (50.8)	970 (51.4)

PMR - Percutaneous myocardial revascularization compared to continued medical therapy in patients with refractory angina [6]	69 (2.4)	52 (2.8)

TMR - Transmyocardial laser revascularization compared to continued medical therapy in patients with refractory angina [5]	200 (7.0)	148 (7.8)

SPiRiT - Spinal cord stimulation (SCS) compared to PMR in patients with refractory angina [16]	76 (2.7)	53 (2.8)

Angina total (PMR, TMR, SPiRiT)	345 (12.1)	253 (13.4)

Total	2855	1887

### Measurements assessed

The EQ-5D questionnaire consists of 5 questions covering health domains of mobility, self-care, usual activity, pain and anxiety/depression [1-3]. Each domain has three levels of severity: no problems, some or moderate problems and severe problems. Utility weights can then be attached to the EQ-5D health state provided by the questionnaire [18]. Utility values range from 1 (best possible health), through 0 (death) to -0.59 (worse than death) [19]. The UK algorithm for calculating the EQ-5D index was used in this study [18].

Total exercise time was available from a modified Bruce protocol treadmill test (ETT). The Bruce protocol requires walking on a treadmill at a given speed and with a given grade, both of which increase through three stages [14,20].

Angina class was measured by the Canadian Cardiovascular Society Angina Scale. The CCS was recorded as a 5-point score according to the amount of exercise required to bring on angina from 0 (no angina even on strenuous or prolonged physical exertion) to IV (angina with minimal exertion or at rest).

The disease-specific Seattle Angina Questionnaire (SAQ) has five dimensions related to angina: the exertional capacity scale (ECS), anginal stability scale (ASS), anginal frequency scale (AFS), treatment satisfaction scale (TSS) and the disease perception scale (DPS). Each scale has a range of 0 to 100 with higher values representing greater functioning/satisfaction and fewer limitations.

### Statistical analysis

Continuous variables were summarized using the mean and standard deviation. Relationships between the EQ-5D index and continuous explanatory variables were explored by studying scatter plots and correlations between the variables. Categorical variables were summarized using frequencies and proportions. The relationship between the EQ-5D index and categorical variables was explored by summarizing the mean and standard deviation of the EQ-5D index for different levels of these variables, and using the Student's t-test or analysis of variance for comparisons.

For mapping, a base linear model was fitted using ordinary least squares (OLS) estimation with EQ-5D index as the dependent variable and age, sex and a proxy for disease stage as explanatory variables in the model using the estimation dataset. The proxy 'disease stage' variable was created by taking into account both the procedures patients had undergone and the time point of the EQ-5D index measurement. Patients were classified as a) having had only medical management (MM, ie. a baseline measurement in a patient with no prior procedures and who was randomised to MM during the study), b) being pre-balloon angioplasty +/- stent (PTCA) (ie. a baseline measurement for a patient who went on to have a balloon angioplasty with or without a stent during the study), c) pre-coronary artery bypass graft (CABG), or d) post-PTCA or e) post-CABG, if the patient had one of these procedures before the study began. This variable constituted a proxy for disease stage because patients that only had medical management were likely to be the least ill, but those that entered a study and then had PTCA or CABG were probably at a more advanced stage of disease upon presentation. Furthermore, if patients had one or more revascularisation procedures before entering the study, they are likely to have even further advanced disease. In a situation where a patient could conceivably fit into two categories, for example, if they had both a PTCA and a CABG before the study, or they had a PTCA before the study but would go on to have a CABG during the study, they were put in the category of the most invasive procedure, for example, post-CABG in the first instance, pre-CABG in the second. For the Percutaneous Myocardial Revascularization (PMR), Transmyocardial Laser Revascularization (TMR) and SpiRiT studies, the interventions were PMR, TMR or spinal cord stimulation (SCS) rather than CABG. These were grouped together with CABG since all of these trials involved patients with angina that was not controlled by medical management and for whom conventional revascularisation (PTCA or CABG) had failed or was not possible. Age, sex and disease stage proxy variables were retained in all models. To this base model ETT, CCS class and individual SAQ scales were each added in a stepwise fashion to the model each as an additional explanatory variable. A range of multiple variable models was constructed using the estimation dataset with a combination of these variables depending upon their importance based on adjusted R^2 ^values. The variable that gave the largest increase in adjusted R^2 ^was added first, and then all remaining variables were tested again one at a time. Variables were added until there was no appreciable change in adjusted R^2 ^(less than 5%). The root mean square error (RMSE) and mean absolute error (MAE) were also calculated to assess model fit and prediction ability [13]. The RMSE was calculated by taking the square root of the mean square error from the models. MAE was calculated as the sum of the absolute differences between the predicted and observed values, divided by the sample size. Adjusted R^2 ^was used for choosing models rather than one of these measures of prediction accuracy because it is penalised for larger models, with the use of the less than 5% change criterion further contributing to a parsimonious model. Only two of the five SAQ scales were available for the Appropriateness for Coronary Revascularization (ACRE) study, so interaction terms were used to examine whether there were differences in the effect of these scales in the ACRE data as compared to the other studies. Interaction terms between ETT, CCS and SAQ and the disease stage proxy variable were also pre-specified. This allowed for different relationships between these variables and the EQ-5D index in different disease stage groups, which was important given that a high degree of heterogeneity in these relationships has previously been shown [14].

One of the multiple variable models was chosen as the mapping model based upon explanation of the maximum amount of variability in the EQ-5D index with the fewest variables, as well as relatively low RMSE and MAE values. To validate this model the regression equation was applied to the data in the validation dataset, predicted values of the EQ-5D index were obtained for each person, and these predicted values compared to the observed values. Standardised residuals and fitted EQ-5D index values from fitting the final model in both the estimation and validation datasets were plotted against one another. A Bland-Altman analysis was performed, both in the estimation and validation datasets, to see how well the observed and predicted EQ-5D index agreed and if there appeared to be any systematic measurement bias in the predicted index. The intraclass correlation coefficient (ICC) for the observed and predicted values was calculated as a further measure of agreement. The final model was also fitted to the data in the validation dataset to obtain the adjusted R^2^, RMSE and MAE.

The study includes secondary analysis of results from a range of studies. All primary studies had ethical approval from Local Research Ethics committees between 1993 and 2001.

## Results

There were 2855 records in the estimation dataset and 1887 in the validation dataset. The estimation and validation datasets had similar distributions of the variables of interest (Tables 2 and 3). The EQ-5D index was slightly higher for men than for women and significantly lower for higher CCS angina classifications (Table 3). The EQ-5D index was also significantly lower in patients that were post-CABG/other serious intervention compared to patients in the other disease stage proxy groups (Table 3). Table 4 shows that the ECS of the SAQ had a marked correlation (correlation coefficient > 0.6) with the EQ-5D index, while most of the other correlations were low or moderate. Age was not correlated with the EQ-5D index in the estimation dataset.

**Table 2:** Summary of continuous variables in estimation and validation datasets

Variable	Estimation dataset sample size	Validation dataset sample size	Estimation dataset mean (SD)	Validation dataset mean (SD)
EQ-5D	2855	1887	0.68 (0.29)	0.67 (0.30)

Age	2855	1887	63.8 (9.7)	64.0 (9.2)

ETT	1356	883	10.1 (4.6)	10.1 (4.4)

SAQ ECS	1119	712	70.4 (24.4)	71.9 (25.4)

SAQ ASS	1812	1200	53.3 (24.5)	53.4 (24.9)

SAQ AFS	2314	1491	74.2 (27.6)	73.7 (28.3)

SAQ DPS	1200	764	62.4 (25.2)	63.7 (25.8)

SAQ TSS	1200	764	88.7 (15.5)	89.2 (14.4)

Key: SD = standard deviation, EQ-5D = EuroQol 5D index, ETT = exercise treadmill time, SAQ = Seattle Angina Questionnaire, ECS = exertional capacity scale, ASS = anginal stability scale, AFS = anginal frequency scale, DPS = disease perception scale, TSS = treatment satisfaction scale

**Table 3:** Summary of categorical variables in estimation and validation datasets

Variable	Estimation dataset, n (%)	Validation dataset, n (%)	Mean (SD) EQ-5D (estimation dataset)	p-value (estimation dataset)
Gender				0.04

Male	2059 (72)	1361 (72)	0.69 (0.30)	

Female	796 (28)	526 (28)	0.66 (0.29)	

CCS class				<0.001

0	499 (18)	319 (17)	0.81 (0.24)	

I	513 (18)	306 (16)	0.78 (0.21)	

II	801 (28)	509 (27)	0.70 (0.23)	

III	364 (13)	252 (13)	0.49 (0.29)	

IV	326 (11)	265 (14)	0.38 (0.33)	

Disease stage proxy				<0.001

MM	1241 (44)	815 (43)	0.71 (0.28)	

Pre PCI	116 (4)	63 (3)	0.77 (0.21)	

Post PCI	428 (15)	259 (14)	0.70 (0.29)	

Pre CABG/SCS/laser	66 (2)	47 (3)	0.76 (0.20)	

Post CABG/SCS/laser	993 (35)	698 (37)	0.61 (0.31)	

Key: SD = standard deviation, EQ-5D = EuroQol 5D index, CCS = Canadian Cardiovascular Society Angina Classification, MM = medical management, PCI = balloon angioplasty ± stent, CABG = coronary artery bypass graft, SCS = spinal cord stimulation, laser = percutaneous or transmyocardial laser revascularization

**Table 4:** Correlation of continuous variables with EQ-5D index from estimation dataset

Variable	Correlation coefficient	p-value
Age, n = 2855	0.05	0.008

ETT, n = 1356	0.42	<0.001

SAQ ECS, n = 1119	0.63	<0.001

SAQ ASS, n = 1812	0.30	<0.001

SAQ AFS, n = 2314	0.45	<0.001

SAQ DPS, n = 1200	0.57	<0.001

SAQ TSS, n = 1200	0.30	<0.001

Key: EQ-5D = EuroQol 5D index, ETT = exercise treadmill time, SAQ = Seattle Angina Questionnaire, ECS = exertional capacity scale, ASS = anginal stability scale, AFS = anginal frequency scale, DPS = disease perception scale, TSS = treatment satisfaction scale

Results of the mapping model constructed from the estimation dataset are described in Tables 5 and 6. There were 1106 records in the estimation data with non-missing covariates in the final model. The variables in the base model - age, sex and disease stage proxy - only explained 4% of the variation in the EQ-5D index and gave an RMSE of 0.288. When either of ETT or CCS alone was added to the base model, this was reduced to 0.226 or 0.249, respectively, and just under 30% of the variability was explained. The addition of the ECS scale of the SAQ to the model accounted for the greatest variability in the EQ-5D index (43%) and gave the lowest RMSE (0.179) of all the variables when added singly. As the ASS and AFS scales were the only SAQ scales available from the ACRE study, and the ACRE data were therefore no longer included in the multiple variable models once the other scales were added, their relationship to the EQ-5D index was compared in ACRE and the other studies using an interaction term. The results for models with ASS and AFS have also been presented with the ACRE data excluded (Tables 5 and 6). The interaction term was significant for ASS, suggesting a different relationship between ASS and EQ-5D index in ACRE as compared to the other studies. There was little difference in the amount of variability explained, by ASS, however, whether ACRE data were included or not. The error was reduced when the ACRE data were excluded. In the case of the AFS scale, the interaction term was not significant. AFS appeared to provide greater error reduction and to explain more variability in the EQ-5D index when the ACRE data were removed. Other interaction terms did not improve the fit of the model appreciably.

**Table 5:** Results of multiple variable modelling in the estimation dataset

Model	1	2	3	4	5	6	7	8	9	10	11	12
Sample size	2844	1345	2492	1108	1801	1186	2303	1186	1189	1189	1106	1104

Age per year	0.002	0.008	0.001	0.003	0.002	0.003	0.0004	0.002	-0.001	0.002	0.002	0.002
	(0.001, 0.004)	(0.006, 0.009)	(0.0002, 0.002)	(0.002, 0.005)	(0.0004, 0.003)	(0.001, 0.004)	(-0.001, 0.002)	(0.001, 0.003)	(-0.002, 0.001)	(0.0004, 0.003)	(0.001, 0.003)	(0.001, 0.003)

Sex = male	0.054	-0.031	0.038	-0.012	0.031	0.034	0.035	0.019	0.013	0.029	-0.009	-0.009
	(0.029, 0.078)	(-0.061, -0.002)	(0.015, 0.061)	(-0.037, 0.012)	(0.003, 0.059)	(0.004, 0.064)	(0.013, 0.058)	(-0.007, 0.046)	(-0.013, 0.039)	(-0.001, 0.059)	(-0.033, 0.014)	(-0.033, 0.014)

MM	0.117	0.111	0.043	0.016	0.123	0.133	0.053	0.049	0.089	0.132	0.021	0.023
	(0.091, 0.142)	(0.079, 0.142)	(0.018, 0.068)	(-0.015, 0.046)	(0.091, 0.154)	(0.098, 0.169)	(0.027, 0.078)	(0.016, 0.083)	(0.057, 0.121)	(0.095, 0.168)	(-0.009, 0.050)	(-0.007, 0.052)

Pre PCI	0.165	0.134	0.107	0.022	0.181	0.130	0.152	0.089	0.117	0.120	0.048	0.047
	(0.110, 0.221)	(0.086, 0.181)	(0.057, 0.157)	(-0.020, 0.064)	(0.127, 0.235)	(0.079, 0.181)	(0.103, 0.200)	(0.043, 0.135)	(0.072, 0.161)	(0.069, 0.172)	(0.008, 0.088)	(0.007, 0.087)

Post PCI	0.101	0.127	0.036	0.027	0.084	0.116	0.037	0.047	0.076	0.129	0.018	0.015
	(0.068, 0.134)	(0.082, 0.172)	(0.004, 0.067)	(-0.013, 0.067)	(0.043, 0.125)	(0.068, 0.165)	(0.006, 0.068)	(0.003, 0.092)	(0.033, 0.119)	(0.080, 0.178)	(-0.021, 0.056)	(-0.023, 0053)

Pre CABG*	0.151	0.146	0.132	0.048	0.178	0.124	0.186	0.120	0.113	0.117	0.073	0.071
	(0.079, 0.223)	(0.087, 0.205)	(0.069, 0.195)	(-0.004, 0.100)	(0.108, 0.248)	(0.060, 0.188)	(0.122, 0.249)	(0.063, 0.177)	(0.057, 0.169)	(0.053, 0.182)	(0.024, 0.123)	(0.022, 0.121)

ETT per minute		0.027										
		(0.024, 0.030)										

CCS**												
0			0.426									
			(0.391, 0.461)									

I			0.395									
			(0.360, 0.430)									

II			0.307									
			(0.274, 0.340)									

III			0.114									
			(0.077, 0.152)									

ECS**				0.0062							0.0036	0.0036
				(0.0057, 0.0066)							(0.0030, 0.0042)	(0.0030, 0.0042)

ASS					0.0032							
					(0.0027, 0.0037)							

ASS (ACRE excluded)						0.0031						0.0004
						(0.0025, 0.0036)						(-0.0001, 0.0008)

AFS							0.0046					
							(0.0043, 0.0050)					

AFS (ACRE excluded)								0.0047			0.0015	0.0014
								(0.0043, 0.0051)			(0.0010, 0.0020)	(0.0008, 0.0020)

DPS									0.0055		0.0021	0.0018
									(0.0050, 0.0060)		(0.0015, 0.0027)	(0.0012, 0.0024)

TSS										0.0043		0.0010
										(0.0035, 0.0052)		(0.0002, 0.0017)

*CABG also includes SCS (spinal cord stimulation) and laser (percutaneous or transmyocardial laser revascularization ) treatments. Reference category - Post CABG.

**Reference category, class IV.

***All SAQ score parameter estimates are for a one point increase in the score for the given scale.

Key: MM = medical management, PCI = balloon angioplasty ± stent, CABG = coronary artery bypass graft, ETT = exercise treadmill time, CCS = Canadian Cardiovascular Society Angina Classification, ECS = exertional capacity scale, ASS = anginal stability scale, ACRE = Appropriateness for coronary revascularization, AFS = anginal frequency scale, DPS = disease perception scale, TSS = treatment satisfaction scale

**Table 6:** Measures of prediction from multiple variable modelling in the estimation dataset

Model	Variables in model (in addition to age, sex and disease stage)	RMSE	MAE	Adjusted R^2^
1	---	0.288	0.209	0.04

2	ETT	0.226	0.169	0.29

3	CCS	0.249	0.185	0.28

4	SAQ ECS	0.179	0.130	0.43

5	SAQ ASS	0.264	0.193	0.13

6	SAQ ASS with ACRE data excluded	0.228	0.163	0.15

7	SAQ AFS	0.243	0.175	0.23

8	SAQ AFS with ACRE data excluded	0.204	0.148	0.32

9	SAQ DPS	0.199	0.144	0.35

10	SAQ TSS	0.230	0.165	0.13

11	SAQ ECS, AFS, DPS	0.170	0.122	0.48

12	SAQ ECS, ASS, AFS, DPS, TSS	0.169	0.121	0.49

Key: RMSE = root mean square error, MAE = mean absolute error, ETT = exercise treadmill time, CCS = Canadian Cardiovascular Society Angina Classification, SAQ = Seattle Angina Questionnaire, ECS = exertional capacity scale, ASS = anginal stability scale, AFS = anginal frequency scale, DPS = disease perception scale, TSS = treatment satisfaction scale

The model equations for the chosen prediction model, Model 11, which has the base variables plus ECS, DPS and AFS of the SAQ is shown below. This model explained 48% of the variation in the EQ-5D index in the estimation dataset and had an RMSE of 0.170. The equation for Model 12, which has all of the SAQ scales included, is also shown. The RMSE for this model was 0.169, so the prediction error from these two models was not appreciably different.

Model 11: EQ-5D index = 0.147 + 0.002*age - 0.009(if male) + 0.021(if MM) + 0.048(if pre-PCI) + 0.018(if post-PCI) + 0.073(if pre-CABG) + 0.0036*(ECS) + 0.0021*(DPS) + 0.0015*(AFS)

Model 12: EQ-5D index = 0.071 + 0.002*age - 0.009(if male) + 0.023(if MM) + 0.047(if pre-PCI) + 0.015(if post-PCI) + 0.071(if pre-CABG) + 0.0036*(ECS) + 0.0004*(ASS) + 0.0018*(DPS) + 0.0014*(AFS) + 0.0010*(TSS)

**Figure 1:**
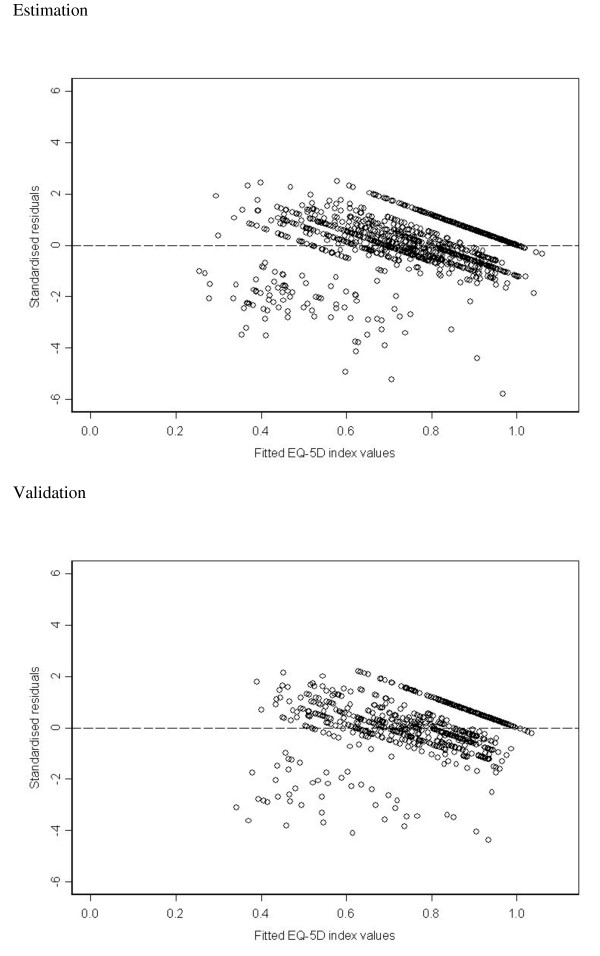
**Plots of standardised residuals against fitted EQ-5D index values for Model 11 in estimation and validation datasets**. Key: EQ-5D = EuroQol 5D index

**Figure 2:**
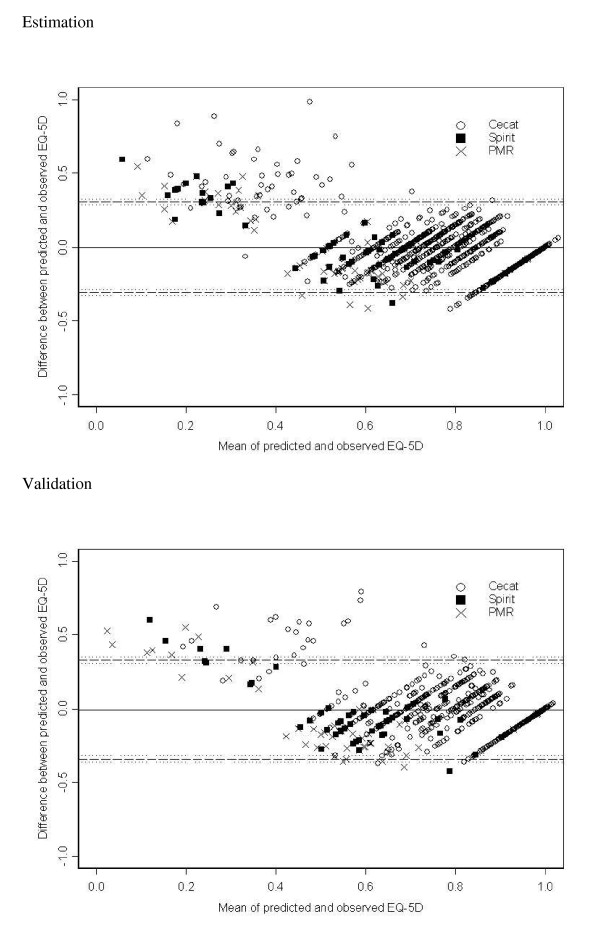
**Bland-Altman plots of agreement between predicted and observed EQ-5D index from Model 11 in estimation and validation datasets**. Key: EQ-5D = EuroQol 5D index, CeCAT = Cost-effectiveness of functional cardiac testing in the diagnosis and management of CHD study, Spirit = Spinal cord stimulation (SCS) compared to PMR in patients with refractory angina study, PMR = Percutaneous myocardial revascularization compared to continued medical therapy in patients with refractory angina study

There were 702 records with non-missing covariates in the final model in the validation dataset. The ICC for the observed and predicted values of the EQ-5D index was 0.64 (95% CI 0.59, 0.68). When the mapping model was applied to the validation dataset it produced an adjusted R^2 ^of 0.44, RMSE of 0.167 and an MAE of 0.123, which were similar to the results in the estimation dataset. Figure 1 shows plots of standardised residuals versus fitted EQ-5D index values in both the estimation and validation datasets, showing evidence of the partly discrete nature of the EQ-5D index at its upper end. The Bland-Altman analysis (Figure 2) shows reasonable agreement for higher values of the EQ-5D index, but poor agreement for people with EQ-5D index values of approximately 0.4 or less in both the estimation and validation datasets. Table 7 shows that an observed EQ-5D of 0.4 or less was associated with a larger RMSE. The lowest predicted value obtained for EQ-5D index in the validation set was 0.25, while the lowest value in the data was -0.24. The 95% limits of agreement in the validation dataset were (-0.34, 0.33). The mean difference between predicted and observed EQ-5D index values for the three trials that measured the covariates in the final model were (predicted - observed): 0.004 (95% CI -0.009, 0.016) for CeCAT, -0.078 (-0.149, -0.007) for PMR and -0.035 (-0.094, 0.025) for SpiRiT.

**Table 7:** Performance of prediction model in estimation and validation datasets by observed EQ-5D level

	Model 11 in estimation dataset	Model 11 in validation dataset
RMSE, n	0.170, 1106	0.167, 702

RMSE - EQ-5D <= 0.4, n	0.130, 86	0.126, 44

RMSE - EQ-5D > 0.4, n	0.110, 1020	0.110, 658

Key: EQ-5D = EuroQol 5D index, RMSE = root mean square error

## Discussion

This study aimed to build a model to map from cardiac patients' demographic and outcome measures to the EQ-5D index. The SAQ ECS was the strongest predictor of the EQ-5D index, and had the lowest RMSE as compared to other variables available. The SAQ DPS and AFS scores also entered the model, indicating that a disease-specific measure of patient health and disease perception was an important predictor of the generic measure of HRQoL. If interest centres on mapping the EQ-5D index in another disease area, disease specific measures for the disease in question may also be important. The mapping exercise was initially performed with the EQ-5D index bounded to a 0-1 scale and logit transformed as the outcome variable for the OLS models. There was little difference in prediction results whether these transformations were applied or not, and so the non-transformed EQ-5D index was used as the outcome for simplicity. The residual plots show some potential difficulties with using OLS (Figure 1). The ceiling effect of EQ-5D index values close to 1 was apparent as well as patterns that are probably partly due to EQ-5D index not taking all values on the continuum. Others have acknowledged this issue and explored other models with similar findings [21]. Tsuchiya et al. mentioned the option of transforming the data, but suggested that it may be less important for prediction as opposed to when modelling for explanatory purposes, and also that transformation may make prediction models less applicable in situations where the distribution of the data may be different [21]. We feel the use of OLS models was reasonable in this study, given that there was a large amount of data available. This suggests that mean values could be assumed to have an asymptotic Normal distribution and unbiased estimators were obtained. Also, we found that the results were robust to the different forms of the outcome variable used.

The final mapping model explained 48% of the variability in EQ-5D index and provided essentially the lowest RMSE at 0.17. This RMSE was, however, higher than the minimal important difference for the EQ-5D index of 0.05 [22,23] and high compared to some RMSEs found in other similar studies of mapping the EQ-5D index [13]. The ICC was consistent with a moderate to good correlation between observed and predicted EQ-5D scores. However, using the model to predict the observed EQ-5D index in the validation dataset did not indicate good prediction on average and the Bland-Altman plot showed that the mapping model over-estimated the EQ-5D index for people with observed values of approximately 0.4 and below in both the estimation and validation datasets. The plot had relatively wide 95% agreement limits of approximately ± 0.3, which again are much larger than the minimal important difference for the EQ-5D index [22,23]. The RMSEs were also higher for EQ-5D <= 0.4 in both datasets. A similar result has been seen before for patients with stable angina, where a model mapping clinical measures on to the EQ-5D index explained 37% of the variability in the EQ-5D index and also performed poorly in individuals with EQ-5D index values of about 0.4 or less [15]. Other studies mapping other HRQoL measures on to EQ-5D have had similar findings [21,24]. One possible reason for the poorer prediction could be sparse data; Table 7 shows there were few people in the data with an observed EQ-5D index of <= 0.4. Using data where there are more patients with low EQ-5D index values might help better predict values across the range.

Several different strategies for improving the predictive ability of the model were explored. These included adding the ETT and CCS variables back into the final model, even though they did not enter the model under the pre-specified criterion. These two variables were tried in the final model as their lack of importance in the mapping model was somewhat surprising. This is perhaps especially true for the CCS, which was found in a previous study to have a strong relationship with the EQ-5D index [14]. These variables did not improve prediction, possibly due to the inclusion of disease stage. A model with higher order SAQ terms was also tested, as were several models with interaction terms between the disease stage proxy variable and the other variables in the final model. Although the model with higher order SAQ scale terms allowed for the prediction of lower values of the EQ-5D index, none of these strategies improved the agreement between the predicted and observed values appreciably. Similar findings have been published in the wider mapping literature [13]. It is possible that an important predictor of HRQoL, such as the patient's social isolation and/or mental state, was missing. Such information might contribute to explaining the difference between two patients with the same level of disease severity but very different EQ-5D index values.

Finally, the mean difference in observed and predicted values was smallest for patients from the CeCAT study, which was the study that contributed the most data and also that had the healthiest participants. This may mean the prediction model derived here is more applicable to patients early in the course of disease and that further study using data with more patients across the spectrum of disease could improve prediction, perhaps especially towards the lower end of the EQ-5D index range. There was a further nuance in prediction of the EQ-5D index between studies shown by these estimates - the prediction model under-predicted values of the EQ-5D index overall in the PMR study, and to some extent in the SpiRiT study - the Bland-Altman analysis shows over-prediction for the few people with an observed EQ-5D index below 0.4 for all three studies, but some under-prediction for people with EQ-5D index measurements of greater than 0.4 in the PMR and SpiRiT studies.

Another potential explanation for the poor prediction is that while the 5-question, 3-response format makes the EQ-5D easy to administer and complete, it describes a relatively small number of possible health states and does not discriminate well, especially towards the end of the scale describing good health [25,26]. In addition, the second level on the EQ-5D (some or moderate problems) could include people with quite a wide range of problems in a given domain, corresponding to widely different levels of HRQoL. A 5-level version of the EQ-5D has been created and piloted and shows evidence of feasibility and greater face validity for patients [27], less of a ceiling effect, and better health state discrimination [27,28]. Besides potentially improving the discriminatory properties of the EQ-5D, the 5-level EQ-5D index will allow for more variability in the measure and may more accurately reflect some health states, possibly making mapping of the EQ-5D index from other measures more successful across all severity levels.

### Limitations

One limitation of the study was that only three out of the five studies that were available had all of the necessary covariates. This limits the external validity of the findings and may have other unknown effects that users of the mapping algorithm should bear in mind. For example, the ACRE study only included two of the five SAQ scales and, when interaction terms were used to compare the effect of these two scales in ACRE versus the other studies, there was some evidence of a difference, meaning the effects might have been different had we had such information from ACRE patients or had data from more studies. Future work should include further development and validation of potential mapping models on datasets with more complete information on covariates and more data on patients with more severe cardiac disease. Secondly, the UK algorithm for calculating the EQ-5D index was used, so the models may not be applicable to cardiac patients from other countries. Thirdly, we have not explicitly accounted for the correlation between baseline and treatment measurements on individuals since baseline measurements will not always be available and the models should only be used for patients with similar clinical and demographic profiles. Future work should include validating the model on an independent sample [13], and for patients with different characteristics, undergoing different cardiological procedures. Validating the model in a completely independent dataset would lend further support to the findings.

## Conclusions

In conclusion, it was possible to construct mapping models for the EQ-5D index using demographic, disease stage and cardiac outcome measures for a group of cardiac patients that performed better in predicting the EQ-5D index for values above 0.4, and less well for values below that level, where the EQ-5D index was over-estimated. The root mean square error derived from fitting the final model in the validation dataset was larger that the minimal important clinical difference for EQ-5D. Prediction of the EQ-5D index is possible, however, due to the relatively poor prediction across the range, inclusion of a preference-based measure in a study where cost-effectiveness analysis is an aim would be a better approach than prediction of the EQ-5D index from other measures.

## Competing interests

The authors declare that they have no competing interests.

## Authors' contributions

KG performed the analysis and drafted and edited the manuscript. MD edited the manuscript. MB designed the study and edited the manuscript. LS designed the study and edited the manuscript.

All authors have read and approved the final manuscript.
